# Magnitude of home delivery and associated factors among child bearing age mothers in Sherkole District, Benishangul Gumuz regional state-Western-Ethiopia

**DOI:** 10.1186/s12889-020-08919-8

**Published:** 2020-05-27

**Authors:** Resom Berhe, Adane Nigusie

**Affiliations:** Department of Health Education and Behavioral Sciences, College of Medicine and Health Science, Institute of public health, University of Gondar, Gondar, Ethiopia

**Keywords:** Home delivery, Women’s in the child bearing age, Sherkole

## Abstract

**Background:**

The World Health Organization estimates that globally only 43% of women have access to skilled care during deliveries and the rest are exposed to unskilled delivery service. A recent Ethiopian Demographic and Health Survey report stated that maternal death was 412 per 100,000 in 2016.This still indicates that maternal health remains a major public health problem in Ethiopia irrespective of the government’s measure to institutional delivery. Therefore, the aim of this study was to assess the magnitude of home delivery and associated factors among women of child bearing age in Sherkole district, Western Ethiopia.

**Methods:**

A community based cross sectional study was conducted among women aged 15–49 years in Sherkole district, Benishangul Gumuz region from January to June 2018. A total of 451 randomly selected women were included in the study. Stratified sampling followed by simple random sampling technique was used to select the study participants. Data were collected using pretested and structured questionnaires. Bivariate and multivariate logistic regression models were fitted to identify factors associated with home delivery among women in the child bearing age. An adjusted odds ratio with a 95% confidence interval was computed to determine the level of significance.

**Results:**

The magnitude of home delivery was 353 (80%) and were assisted by non-skilled birth attendants. Mothers whose husband chooses the place of delivery [AOR: 5.6, 95% CI (2.1–15.2), Mothers’ occupation ([AOR: 0.21 95% C I (0.08–0.57), ANC visit [AOR: 95 CI: 5.1(1.6–15.8), decision making [AOR: 95 CI: 0.3(0.01–0.7)] and traditional remedies [AOR: 95%CI: 0.03 (0.01–0.09)] were significantly associated with home delivery.

**Conclusions:**

Based on the findings of the survey, it was concluded that the overall magnitude of home delivery was found to be high. Therefore, it is recommended that the promotion of antenatal care follow-up with maternal and child health information particularly on delivery complications or danger signs needs due attention and remedial actions. In addition, it is indispensable introducing defaulter tracing mechanisms in ANC services, by learning from experiences of settings that have already adopted it.

## Background

Home delivery is a place where women deliver outside the health facilities, give birth at home, where risks of mortality and sepsis are the cause of many complications, which may lead to maternal death [1]. In most cases, home delivery is practiced without the presence of qualified personnel [1]. Delivery assisted by qualified personnel (skilled birth attendant) is recognized to contribute to the healthier outcome of pregnancy and child birth during delivery. In fact, in developing countries, a significantly high percentage of deliveries are made by traditional birth attendants (TBA) [2].

It is acknowledged that maternal deaths have been shown to subsidize to opposing perinatal outcomes such as stillbirths and interventions to decrease stillbirths are likely to reduce maternal mortality too [3]. Similarly, majority of the deliveries that take place at home attended by traditional birth attendants in developing countries have been shown to be unable to subsidize to the reduction of maternal mortality [4].

On the global scale, home deliveries in the developed western countries constitute a bordering share of total deliveries being mainly below 2% with the exception of The Netherlands and Malawi where home deliveries are 12.9% and above 30% [5, 6]. It has also been noted that maternal deaths happening worldwide has been estimated at 358,000 declines from the previous high of 529,000 in the recent past [3]. However, levels of these deaths in unindustrialized countries is too high (99%) and is ordered among the highest in sub-Saharan African region, estimated at 640 per 100,000 live births, followed by South Asia which had an estimated 280 deaths per 100,000 live births in 2015 [7].

Worldwide, one-third of births take place at home in the absence of professional attendants. It has been estimated that less than 50% of births in Africa are attended by skilled health workers [6, 7]. Ethiopian Demographic and Health Survey report (EDHS) stated that maternal death was 412 per 100,000 in 2016. This still indicates that maternal health remains a major public health problem in Ethiopia. Irrespective of the government’s measures to institutional delivery assisted by skilled attendants, home delivery remains high, estimated at over 79% of all pregnant women [8].

In Ethiopia, antenatal care coverage is 62%, implying that women are aware of the importance of attending a clinic but only a few deliveries take place in the health facility. A skilled attendant at deliveries is estimated at 28% nationally and lower in rural areas. Even though access to a health facility in the country is good with over 85% of the population living within 3 km of primary health care or outreach health post and over 97% of the population within 5 km but sadly, a very low proportion of the women uses the health facilities for delivery [8].

Various factors were reported to be associated with home delivery among pregnant women. Among these are low socioeconomic status [9–16], the lower chance of being educated, lack of pregnancy monitoring [17–20]. In addition, poor accessibility to health facilities [21–24] and limited female involvement in decision making power [25–31] are key factors for low service uptakes. Therefore, accurate epidemiological information is necessary to understand the magnitude of home delivery among women of child bearing age, to guide interventions that help to improve institutional delivery and improve women wellbeing and to monitor trends over time. Thus, the aim of this study was to assess the magnitude of home delivery and associated factors among women of child bearing age in Sherkole district, Western Ethiopia.

## Methods

### Study design

Community based cross sectional study was carried out from February – March 2018 in Sherkole district, western Ethiopia.

### Study area

Sherkole district is found 782 km away from Addis Ababa, the capital city of Ethiopia. According to the population projection for 2017, the total population size of the district was estimated to be 35,542 of which 18,616 are female. The district is organized into 19 Kebeles (the lowest administrative unit) and of these 14 of the kebeles are found in the rural areas. As to the health service facilities in the district; there were one district hospital, two health centers, thirteen health posts providing health care services. During rainy seasons most parts of the districts become inaccessible by motor vehicles due to muddy roads.

### Sample size determination and sampling

The sample size was calculated using a prevalence of home delivery 84% in Ethiopia [28] of single sample proportion, 95% confidence level, 5% marginal error, 10% non-response rate and a design effect of 2. The estimated sample size was 451 subjects. This is larger than the total sample size calculated for determinants of home delivery (level of education, place of residence, and number of ANC visits). Hence, 451 was considered to be a sufficient sample size for this study. A multistage probability sampling technique was used to select study participants.

### Study population

The study population comprised of women of child bearing age from 15 to 49 years that gave birth at least once in the last 2 years preceding the survey irrespective of the outcome of the birth and residing in the district for more than 6 months. If women had more than one live birth in the past 2 years, only care received for the most recent birth was considered. Mothers with very sick children were excluded from the study. The population of females in the reproductive age (15–49 years) in the district was 10,030 (53.9%) in 2017 [32].

### Data collection tools and procedure

Data were collected using a pre-tested, structured questionnaire adapted from different works of literatures [33–35] to collect socio-demographic and other relevant reproductive health information that included institutional service utilization and delivery related information of the women. The questionnaire was prepared in English and translated into each local language the participants spoke: Amharic and Bertha. Ten experienced diploma holder nurses who are fluent in speaking both Amharic and Bertha language were recruited and trained for 2 days about the purpose of the study and on the overall data collection procedures. To assure the quality of the data, the questionnaire was then pre-tested by trained data collectors on 5% of the households near adjacent districts, and appropriate modifications were made accordingly. The experience gained in the pretesting was used in organizing the study properly. During the data collection, facilitators were supervised at each site.

### Data management and analysis

The collected data were computerized using Epi-Info 7 and analyzed using SPSS version 20. Both descriptive and analytical statistical procedures were utilized. The study employed descriptive statistics (counts and percentages) for the presentation of demographic data.

Binary logistic regression was used to identify factors associated with home delivery among the mothers in a child bearing age. Variables with P-value less than or equal to 0.25 were fitted into multiple logistic regression models for controlling the possible effect of confounders and finally the variables which had independent association with home delivery were identified on the basis of OR, with 95%CI and p-value less than 0.05.

## Result

### Socio economic and demographic characteristics of the respondent

From a total of 451 mothers who were identified for the study, 441 participated in the study while 10 refused to participate, yielding the response rate of 98%. The mean age of the respondents was 26.8 (SD ± 4.6) years. Higher percentages of the respondents, 438 (99.3%) were from a rural area and almost all of the respondents 434 (98.6%) were Muslims by religion. Regarding the ethnicity, the majority of the respondents 433 (98.1%) were Bertha (see Table 1).

**Table 1:** Socio- economic and demographic characteristics of reproductive age group mothers in Sherkole district Feb-March, Western Ethiopia 2018

Variable	Frequency	%
Age		
15–24	97	23.9
25–34	307	67.9
35–49	37	8.2
Residence		
Rural	343	77.7
Semi urban	98	22.2
Ethnicity		
Berta	433	98.1
Other	8	1.9
Marital status		
Married	417	94.6
Unmarried	24	5.4
Religion		
Muslim	434	98.6
Others	7	1.3
Respondent occupation		
House wife	139	31.6
Farmer	302	68.4
Occupation of the spouse		
Farmer	416	94.3
Others	19	4.3
Respondents educational status		
Illiterate	190	43.7
Able to read and write	41	9.3
Primary school and above	210	47.9
Educational status of spouse		
Illiterate	172	39
Able to read and write	68	15.4
Primary and above	201	45.6
Monthly income (ETB)		
< 320	348	78.9
320–600	93	21.1

### The magnitude of home delivery

The magnitude of Home delivery was 353 (80%) and assisted by non-skilled birth attendants. From these, about 345 (79.7%) were assisted by traditional birth attendants, and the rest by other relatives. From total of 441 respondents, more than half of the respondents 235(53.6%) attended at least one antenatal visit during their last pregnancy.

### Factors associated with home delivery

Variables such as availability of drug and supply, distance to a health facility, ANC visit, traditional remedies, house hold income, occupation of mother, parity, gravidity, husband choice of delivery place and information about the benefit of delivery in a health facility and decision making were candidate by their P-value < 0.25 and entered to multi-logistic regression for their significance. From these factors, ANC visits, occupation of mothers, traditional remedies, and decision-making power were statistically significant.

Mothers’ occupation was found to be a predictor of home delivery, mothers who were farmers by their occupation were about 79% less likely to deliver at home compared with mothers who were house wife [AOR: 0.21 95% CI: 0.21 (0.08–0.57). Mothers who do not attend ANC visit were five times [AOR: 95%CI: 5.1(1.6–15.8)] more likely to give birth at home as compared with mothers who attend ANC. Mothers who decided with their spouse about the place of delivery were about 30% less likely to deliver at home compared to those who decided by their own [AOR: 95%CI: 0.7(0.2–2.1)].

Mothers who don’t prefer traditional remedies were about 97% less likely to deliver at home compared to those who prefer traditional remedies [AOR: 95%CI: 0.03(0.01–0.09)] (see Table 2)*.*

**Table 2:** Factors associated with home delivery among women who gave birth in the last 2 years in Sherkole District, Feb-January, Western Ethiopia, 2018

Home Delivery	Home	Institution	COR	AOR	*P*-value
Distance to health facility					
<2Km	135 (82.80)	28 (17.20)	1.93 (0.95–3.92)	0.8 (0.32–2.01)	0.63
2—5Km	178 (80.21)	44 (19.81)	1.62 (0.83–3.15)	0.9 (0.22–3.71)	0.94
> 5Km	40 (71.41)	16 (28.62)	1	1	
Age category					
15–24	84 (81.00)	20 (19.01)	1.08 (1.51–1.72)	1.6 (1.50–2.03)	0.33
25–34	238 (80.11)	60 (20.11)	1.02 (1.63–5.81)	1.2 (1.20–6.02)	0.82
35 and above	31 (88.61)	8 (11.22)	1	1	
ANC Visit					
Yes	185 (71.111)	76 (28.90)	1		
No	166 (93.32)	12 (6.70)	5.6 (2.65–10.70)	5.1 (1.62–15.8) 1	0.005*
Mother occupation					
House wife	103 (74.11)	36 (25.93)	0.53 (0.32–0.86)	0.21 (0.08–0.57)	0.002*
Farmer	250 (84.50)	46 (15.52)	1	1	
Health Provider Behavior					
Good	108 (77.11)	32 (22.99)	0.77 (0.46–1.26)	1.7 (0.63–4.80)	0.287
Poor	245 (81.40)	56 (18.66)	1	1	
Husband choice					
Institution	72 (31.61)	9((4.52)	1	1	
Home	156 (68.40)	190 (95.51)	0.102 (4.71–20.13)	5.6 (0.11–10.23)	0.12
Traditional Remedy					
No	81((50.91)	78 (49.11)	0.04 (0.02–0.08)	0.03 (0.01–0.09)	0.001*
Yes	272 (96.50)	10 (3.53)	1	1	
Maternal income (ETB)					
< 320	302 (86.54)	46 (13.53)	1	1	
320 and above	51 (54.01)	42 (45.51)	0.19 (0.11–0.31)	0.6 (0.22–1.51)	0.262
Parity					
1	13 (68.41)	6 (31.00)	1	1	
2–5	151 (75.10)	50 (24.91)	1.4 (0.51–3.92)	0.19 (0.008–4.21)	0.290
> 5	189 (85.50)	32 (14.52)	2.7 (0.96–7.73)	0.99 (0.03–11.81	0.993
Gravidity					
1	11 (57.11%)	8 (42.12%)	0.25 (0.91–6.32)	3.4 (0.21–10.23)	0.386
2–5	137 (76.50)	42 (23.51)	0.60 (1.51,10.44)	1.7 (0.08–9.93)	0.725
> 5	205 (84.4)	38 (15.62)	1	1	
Benefit of institution delivery					
Yes	276 (78.00)	75 (21.40)	1	1	
No	77 (85.60)	13 (14.40)	1.61 (0.85–3.06)	2.8 (0.72–11.61)	0.153
Availability of drug					
Good	76 (76.50%)	24 (24.20)	0.68 (0.40–1.18)	0.46 (0.15–1.42)	0.177
poor	264 (82.00%)	58 (18.11)	1	1	
Decision making					
Me	191 (88.00)	24 (11.31)	1		
Husband	86 (82.71)	18 (17.31)	0.6 (0.57–0.92)	0.3 (0.28–1.73)	0.07
Both wife and husband	76 (62.31)	46 (37.71)	0.2 (0.11–0.40)	0.7 (0.21–2.11)	0.049*

## Discussion

The present study revealed the magnitude of home delivery among women in the reproductive age group who gave birth in the preceding 2 years. ANC visit, occupation of mothers, traditional remedies and decision-making power were significantly associated with home delivery.

In the current study, 80% of study participants delivered their child at home and were assisted by non-skilled birth attendants which were comparable to the studies conducted in Kenya [17], Gozamin district of Gojjam, Ethiopia [36], where 67.7 and 75.3% of women gave birth at home.

On the other hand, this result is higher than the results of other studies conducted in Oromia region (58%) [15], Amhara (31%) [20], Malawi (29%) [18], Nigeria (31.5%) [37], Ghana (48%) [38] and Tanzania (44%) [23] . The difference of the result of the present study from those of the previous studies could be attributed to the socioeconomic and geographical variations. However, nearly a similar magnitude was reported in Awi zone (84%) [28].

In this particular study; mothers’ occupation was found to be a predictor of home delivery. Mothers who are farmers were two times less likely to give birth at home compared to those who were house wife [AOR: 0.21 95% CI: 0.21 (0.08–0.57). This is supported by studies conducted in Zambia and Senegal [29, 31]. This could be due to the fact that mothers who engage in agriculture (farming) activities may have their own product and income that can make them economically empowered and hence may confers little decision-making power. Hence, empowering women may serve as a reinforcer for health facility birth.

As to the finding of this study, ANC visit was found significantly associated with home delivery. Mothers who do not attend ANC visit were five times [AOR: 95%CI: 5.1(1.6–15.8)] more likely to give birth at home as compared with mothers who do attend ANC visit. The finding is in line with the studies done in Oromia regional state [26], Tanzania [23], and Nigeria [39]. This could be explained due to nearly the same socio-economic status among sub-Saharan African countries. In addition, poor communication and counseling about the condition of their babies and themselves may also be an additional factor which favors them in experiencing home delivery, but the opposite is true for those who do attend ANC. However, some studies claim that; the frequency of ANC visits during pregnancy would have negative effect in delivering through the assistance of skilled birth attendants as women who are told their pregnancy is normal may feel encouraged to deliver at home [27].

The study also found that home delivery was significantly associated with decision making. Mothers who decide with their husband for the place of delivery were less likely to give birth at home compared to those who decide by themselves. This finding is supported by other studies done in Zambia and Senegal [29, 31]. This could be due to the fact that mothers who make decisions with their husband have highest self-confidence and transparency, and these self-confidences may give them equal opportunity and help them to exercise their right of equality. But most of the time especially in cultural society, majority of women requests permission from their husbands and relatives to go to health facility, which has been described in many studies conducted in African countries.

Furthermore, traditional remedies were also found to be another determinant factor for home delivery. Mothers who don’t prefer traditional remedies were less likely to deliver at home compared to those who prefer traditional remedies. This study was in line with the study conducted in northern part of Ethiopia in Tigray region [12], illustrating that many mothers perceive and belief pregnancy and child birth as a natural gift from God and most of the time ends up with short and easy deliveries, even the one who is in neighboring without hearing that the women is in labor. In line with this, there is a cultural belief regarding the pregnant women that blessing her to end in good outcomes. A study among women in Nigeria also came up with the same finding [37]. This could be explained by under development of modern medical management in Africa and medicalization of western country over African led them to focus on herbal and traditional remedies and healing. Furthermore, the possible reason for mothers to choose traditional remedies is not only about perception, culture and believe but also it is related to in accessibility, affordability and inequity in health coverage and service across the country.

### Limitation of the study

A cross sectional nature of the study does not allow establishing causality of associations and the results should be interpreted cautiously. Recall bias cannot be ruled out about events that took place further from the period of data collection. Social desirability bias may also be a problem.

## Conclusion

Based on the findings of the survey, it was concluded that the overall magnitude of home delivery was found to be high. Therefore, it is recommended that the promotion of antenatal care follow-up with maternal and child health information particularly on delivery complications or danger signs needs due attention and remedial actions. In addition, introducing and adoption of defaulter tracing mechanisms in ANC services at all levels of health services delivery should be an area of focus.

## Data Availability

The datasets used and/or analyzed during the current study are available from the corresponding author on reasonable request.

## Abbreviations

ANC: Antenatal care
ETB: Ethiopian Birr
TBA: Traditional birth attendant
